# Editorial: The role of culture in human thinking and reasoning

**DOI:** 10.3389/fpsyg.2022.1018392

**Published:** 2022-09-13

**Authors:** Hiroshi Yama, Niall Galbraith, Jean Baratgin, Hirofumi Hashimoto

**Affiliations:** ^1^School of Literature and Human Sciences, Osaka Metropolitan University, Osaka, Japan; ^2^Faculty of Education Health & Wellbeing, School of Psychology, University of Wolverhampton, Wolverhampton, United Kingdom; ^3^Laboratory Cognition Humaine et Artificielle, Université Paris 8, Paris, France

**Keywords:** culture, cultural difference, thinking, reasoning, dual-process theory

There have been many studies describing cultural differences in thinking and reasoning. This scientific development is mostly based upon the contrasts between Westerners' analytic cognition and Easterners' holistic cognition (e.g., Nisbett et al., 2001) and/or Westerners' linear thinking and Easterners' dialectical thinking (e.g., Peng and Nisbett, 1999). These studies have come from both social psychologists and cognitive psychologists. Although the former have tried to explain the differences in the frame of social and/or cultural systems, the latter have tried to focus on the cognitive process, which is likely to be influenced by cultural practice. Current studies on the relationship between human thinking and culture from both sides do not necessarily conduct cross-cultural comparisons, but focus on how a culture shapes people's thinking style and how people's thinking and reasoning can be adaptive in each culture.

There have been many explanations for cultural differences in cognition. For example, Miyamoto (2013) identified three levels of cultural differences: distal-level situational factors, proximal-level situational factors, and the psychological level. Cultural differences in thinking and reasoning are said to be at the psychological level. According to her, socio-ecological factors and cultural traditional factors at the distal-level may influence people's thinking and reasoning *via* proximal-level factors. This idea gives us a basic frame of explanation for cultural differences.

Bentahila et al. reviews the literature on moral systems and human moral judgment which are influenced by history, religious beliefs, social ecology, and institutional regulations. Each factor can be either at the distal-level or at the proximal-level. Zhou and Li reports on the influence of the Chinese traditional thought of Zhongyong on resilience. Chun-ling reports an ecological cognitive analysis of Chinese harmonious discourse. Baratgin et al. report on how Kanak's social norms influence people's responses using Knetsch's exchange paradigm. Shao et al. tested the Sapir-Whorf hypothesis considering the difference between French and Chinese languages—they did not observe the influence of language difference and hence they rejected the hypothesis and argue for cultural universality.

Secondly, it is noteworthy that four papers based on dual-process approaches (e.g., Evans, 2010) are published in this special topic. This approach supposes two kinds of process: The intuitive process and the reflective process. Among the cognitive theories of human reasoning, Yama (2018) argued that the dual-process approach is the most promising to be applied to explanations for cultural differences in thinking and reasoning. Dual-process theories make it possible to discuss the influence of explicit/implicit distinctions in cultural practices pertaining to two kinds of rationality: evolutionary adaptation of the intuitive process and normative rationality of the reflective process. Cultural effects have been regarded as implicit (intuitive) hence it is assumed that people's thinking is influenced by cultural products implicitly.

The paper of Suzuki et al. reports the power of implicit process. In spite of people's unconsciousness of cultural context, it still, in effect, influences people's thinking. This proposal is added to argue that intuitive processes can be rational in a sense. Hashimoto et al. test a dual-process model for cultural content: a moral dilemma. They discuss this in the frame of human adaptation. Meada et al. adopt a dual-process approach to the case of punishment and reward. The paper of Majima et al. shows cultural differences in the use of analytical thinking between Westerners and Easterners.

In what direction are studies on the relation between culture and human thinking headed? As categorized into the explanations for cultural differences and the adoption of dual-process theories in this editorial, we propose two directions. One is to pursue the explanations for contemporary cultural diversity and locate these in the frame of “big human history.” In this case, it is necessary for psychologists not only to conduct empirical studies but to access the big data used by historians. The other is to adopt the dual-process approach. This not only gives us the implicit/explicit distinction of cultural influences but introduces the view of human cultural adaptation into research in this field.

## Author contributions

All authors listed have made a substantial, direct, and intellectual contribution to the work and approved it for publication.

## Funding

This research was supported by a grant-in-aid from the Japanese Society for the Promotion of Science (No. 18K03010).

## Conflict of interest

The authors declare that the research was conducted in the absence of any commercial or financial relationships that could be construed as a potential conflict of interest.

## Publisher's note

All claims expressed in this article are solely those of the authors and do not necessarily represent those of their affiliated organizations, or those of the publisher, the editors and the reviewers. Any product that may be evaluated in this article, or claim that may be made by its manufacturer, is not guaranteed or endorsed by the publisher.
